# Plate osteosynthesis combined with bone cement provides the highest stability for tibial head depression fractures under high loading conditions

**DOI:** 10.1038/s41598-022-19107-6

**Published:** 2022-09-14

**Authors:** Philipp Heilig, Lars-Christopher Faerber, Mila M. Paul, Eva Kupczyk, Rainer H. Meffert, Martin C. Jordan, Stefanie Hoelscher-Doht

**Affiliations:** 1 Department of Trauma, Hand, Plastic and Reconstructive Surgery, University Hospital of Wuerzburg, Oberduerrbacher Strasse 6, 97080 Wuerzburg, Germany; 2 Center for Orthopedics and Trauma Surgery, University Medical Center of the Johannes Gutenberg University Mainz, Langenbeckstraße 1, 55131 Mainz, Germany

**Keywords:** Medical research, Materials science

## Abstract

Older patients sustaining tibial head depression fractures often cannot follow the post-operative rehabilitation protocols with partial weight-bearing of the affected limb, leading to osteosynthesis failure, cartilage step-off and arthritis development. Therefore, the aim of this study was to analyse the biomechanical performance of different types of osteosyntheses alone and in combination with bone cement simulating cyclically high loading conditions of tibial head depression fractures. Lateral tibial head depression fractures (AO: 41-B2.2; Schatzker type III) were created in synthetic bones and stabilized using three different osteosyntheses alone and in combination with a commonly used bone cement (chronOS™): 2 screws, 4 screws in the jail technique and a lateral angle-stable buttress plate. After fixation, the lateral tibial plateau was axially loaded in two, from each other independent testing series: In the first test protocol, 5000 cycles with 500 N and in the end load-to-failure tests were performed. In the second test protocol, the cyclic loading was increased to 1000 N. Parameters of interest were the displacement of the articular fracture fragment, the stiffness and the maximum load. The osteosyntheses revealed a higher stiffness in combination with bone cement compared to the same type of osteosynthesis alone (e.g., 500 N level: 2 screws 383 ± 43 N/mm vs. 2 screws + chronOs 520 ± 108 N/mm, increase by 36%, p < 0.01; 4 screws 368 ± 97 N/mm vs. 4 screws + chronOS 516 ± 109 N/mm, increase by 40%, p < 0.01; plate: 509 ± 73 N/mm vs. plate + chronOs 792 ± 150 N/mm, increase by 56%, p < 0.01). Bone cement reduced the displacement of the plate significantly (500 N level: plate: 8.9 ± 2.8 mm vs. plate + chronOs: 3.1 ± 1.4 mm, reduction by 65%, p < 0.01; 1000 N level: 16.9 ± 3.6 mm vs 5.6 ± 1.3 mm, reduction by 67%, p < 0.01). Thus, the highest stiffness and lowest displacement values were found when using the plate with bone cement in both loading conditions (500 N level: 2 screws + chronOs 3.7 ± 1.3 mm, 4 screws + chronOs 6.2 ± 2.4 mm; 1000 N level: 2 screws + chronOs 6.5 ± 1.2 mm, 4 screws + chronOs 5.7 ± 0.8 mm). From a biomechanical perspective, plate osteosynthesis of tibial head depression fractures should always be combined with bone cement, provides higher stability than 2-screw and 4-screw fixation and is a valid treatment option in cases where extraordinary stability is required.

## Introduction

Tibial head fractures account for 10% of all fractures in older patients^1,2^. Depression fractures of the tibial plateau, alone or in combination with a split fracture, occur in younger people in high velocity traumata, whereas in older people they typically occur in low energy traumata due to osteoporosis^1^. The lateral tibial plateau is more frequently affected than the medial tibial plateau. Regularly, operative treatment is needed to restore the articular surface and prevent early arthritis developing by remaining intraarticular steps. After stabilisation of the tibial head depression fractures, postoperative treatment includes partial weightbearing with 15–20 kg for 3 months^1^. Older patients often cannot follow these postoperative treatment instructions and load the leg much more than 20 kg seen in daily clinical practice^3^. Moreover, recent studies trend to recommend early full weight bearing^4^. But higher loading of the tibial plateau cause complications such as early secondary loss of reduction and valgus/varus deformation of the leg axis^5,6^. Therefore, high stability is demanded from the stabilisation technique.

Possible osteosynthesis options for tibial head fractures are screw osteosyntheses with 2 screws or 4 screws placed at a 90° angle to each other; known as a jail or raft technique^7,8^. Plate osteosyntheses are also used to stabilise tibial head fractures. The implants are often combined with a crest bone graft or a bone cement to fill up the remaining metaphyseal bone defect after the reduction. Scanning the literature, there is a lack of analysis of different treatment options under high loading conditions. A study systematically comparing screw and plate osteosyntheses to each other under high loading of the tibial plateau has not yet been published. The aim of this study was to investigate the influence of the type of osteosynthesis, alone and in combination with bone cement, on the secondary subsidence under high loading in pure lateral tibial head depression fractures. We hypothesised that for the plate osteosynthesis a lower subsidence of the cartilage/subchondral bone under loading, compared to screw osteosyntheses, will be found. From the results of the study, we expect particularly important information, which treatment option is biomechanically the most meaningful in clinical cases, when patients are unable to maintain postoperative partial weight-bearing.

## Materials and methods

### Specimen and fracture generation

**Figure 1:**
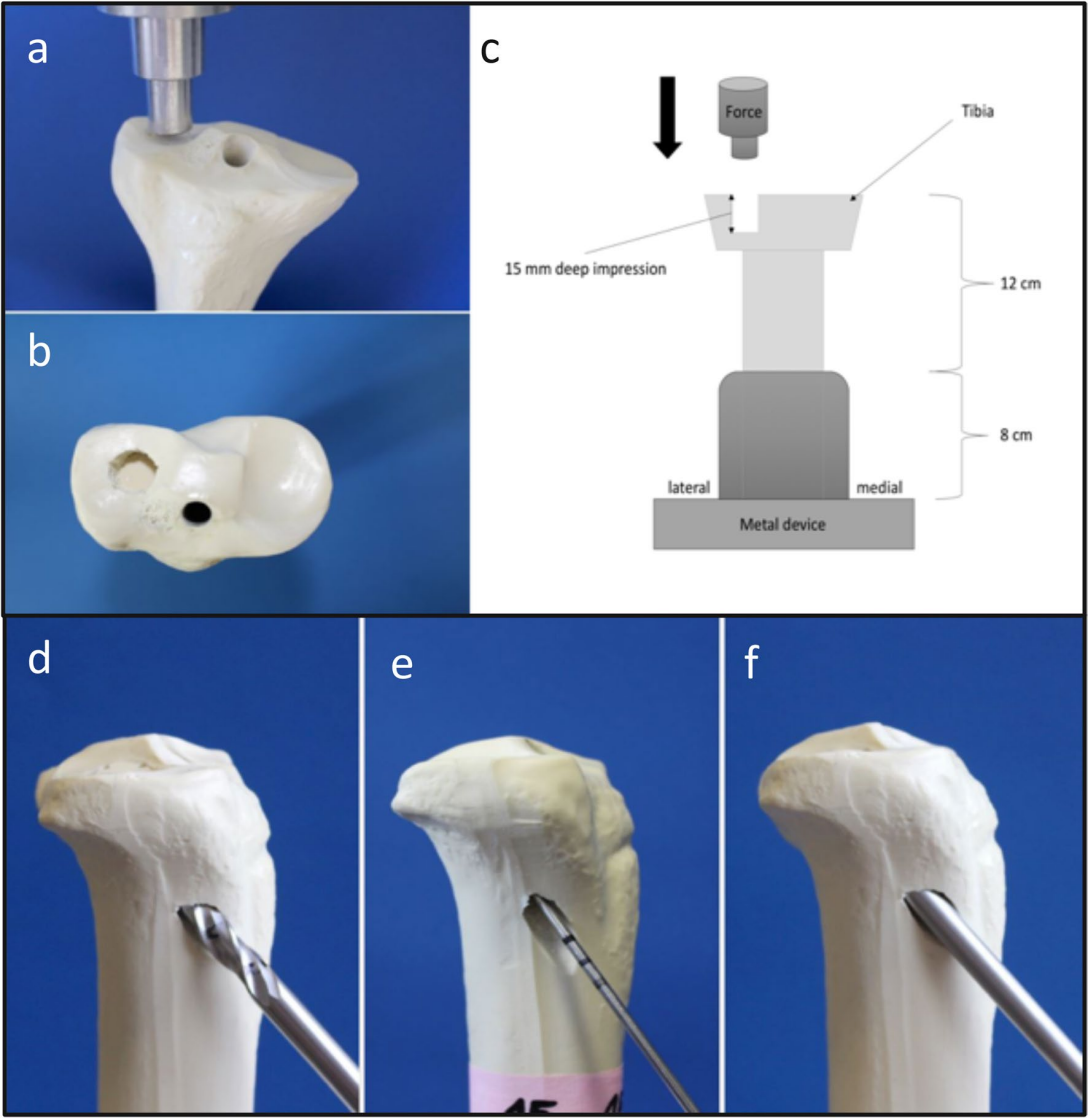
Fracture generation, reduction and test set up are shown. The lateral tibial plateau was loaded axially as demonstrated for fracture generation (**a+b**) and during the biomechanical testing after fixation (**c**). The simulated lateral depression fractures (**a+b**) were reduced anatomically by an approach at the lateral metaphysis (**d**), the depressed articular fracture fragment was detected by a k-wire (**e**) and then elevated by a cannulated ram (**f**).

In total, 126 synthetic tibiae (Synbone 1110, SYNBONE^®^, Switzerland) were tested. These are manufactured with polyurethane foam comprising of a hard cover and a soft inlay structured similar to cortical bone and cancellous inner bone. The cortical thickness at the lateral side of the tibial plateau was 4 mm and at the shaft 3 mm. The width of the tibial plateau amounted to 74 mm and the cortical/cancellous bone model was produced for external fixation methods like used in this study. Previous studies have confirmed that these synthetic bones have comparable biomechanical properties to osteoporotic bones of elderly humans^9,10^. The diaphysis was shortened to 20 cm and embedded in a device at 5° valgus angulation^11,12^. Five 2 mm-holes of 10 mm depth were arranged in a 12 mm circle as breaking points and depression fractures of the lateral plateau with 15 mm depth were reproducible generated by an indentor (Fig. 1a + b). Pure lateral tibial head depression fractures (AO: 41-B2.2; Schatzker type III) were macroscopically and radiographically confirmed, and thereby the rarely occurring split depression fractures were excluded.

### Experimental groups and fixation methods

**Figure 2:**
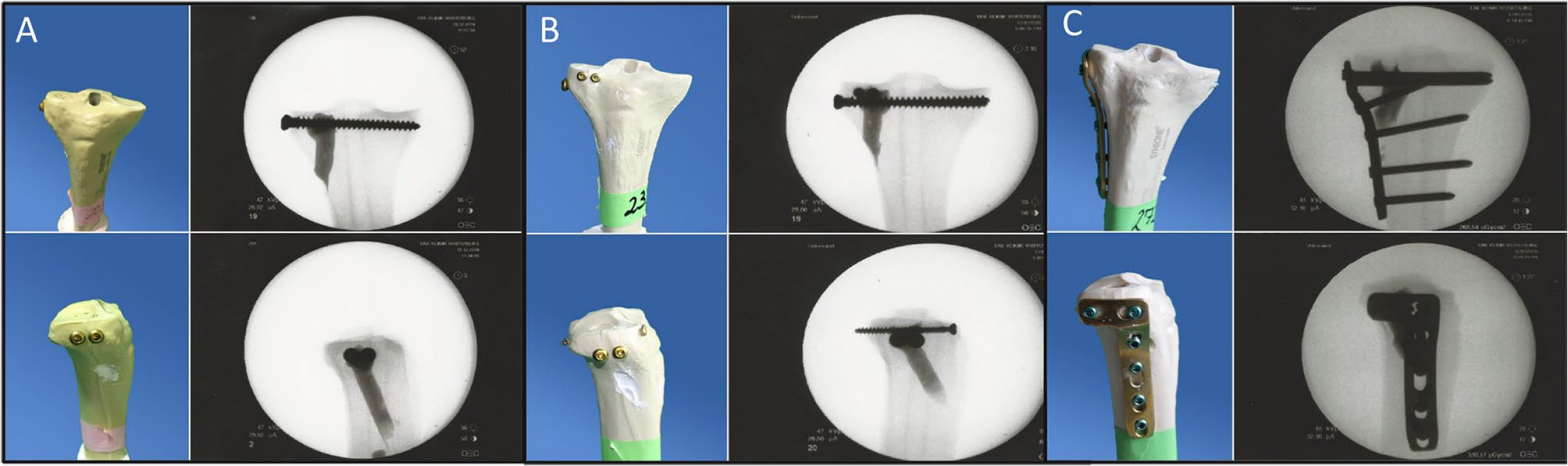
Overview of the different treatment groups. After reduction, the lateral tibial depression fractures were stabilized with three different types of osteosyntheses alone and in combination with bone cement: 2 screws (**A**), 4 screws in the jail or raft technique (**B**) and lateral angle-stable buttress plate (**C**). Specimens and the corresponding x-rays before testing are demonstrated for every osteosynthesis group with bone cement.

The lateral tibial plateau was restored anatomically elevating the depressed fracture fragment like in clinical practice by the minimal invasive arthroscopic reduction and internal fixation (ARIF) technique^13^: The lateral cortical bone of the proximal tibial shaft was opened (Fig. 1d), the depressed fracture fragment detected by a k-wire (Kirschner-wire, controlled under X-ray) (Fig. 1e) and then restored anatomically by a cannulated ram (Fig. 1f). Through the channel left by the cannulated ram, the metaphyseal defect was filled with calcium phosphate cement (chronOS™) only in the plus cement groups. In the osteosyntheses only groups, the defect was left empty. The bones were then randomly assigned to fourteen groups (group size: n = 9; total number of specimens n = 126) (Table 1). We investigated three different types of osteosyntheses: 2 lateral to medial spongiosa 6.5 mm screws, 4 screws in the jail technique (2 lateral to medial spongiosa 6.5 mm screws, 24.0 mm cortex screws placed from anterior to posterior) and a lateral angle-stable buttress plate (fixed with 5.0 mm locking screws, Depuy Synthes, Johnson&Johnson Medical GmbH, Norderstedt, Germany), alone and in combination with a commonly used calcium phosphate cement ChronOS™ Inject (Fig. 2). The self-tapping screws were predrilled like in usual orthopaedic practise. Bone cement only was our control group. The seven possible types of osteosyntheses were then tested in two different biomechanical test set-ups (see below). This resulted in a group total of 14.

**Table 1:** The fourteen test groups are shown: Seven different stabilization techniques were tested in two different cyclic loading protocols, one with 500 N loading and one with 1000 N loading.

Group 500 N level	Osteosynthesis	Bone cement	Group 1000 N level
1	–	ChronOs™	8
2	2 Screws	–	9
3	2 Screws	ChronOs™	10
4	4 Screws	–	11
5	4 Screws	ChronOs™	12
6	Buttress plate	–	13
7	Buttress plate	ChronOs™	14

All osteosyntheses were performed by the same experienced senior orthopaedic surgeon. Bones were placed in an incubator at 37° for 24 h for cement curing. For the same test conditions, also the bones without cement were incubated. Fracture reduction, position of the osteosynthesis and cement replenishment were radiographically controlled.

### Biomechanical test set-up

All tests were conducted using a material testing machine Zwick Roell Z020 (Zwick GmbH & Co. KG, Ulm, Germany). As in previous biomechanical studies, we used axial loading as it was identified to be the main force acting on what during normal daily activity^14–17^. The same indentor used for fracture generation was utilized to apply the load precisely over the reduced fracture fragment (Fig. 1c). The indentor was placed on the lateral plateau and before starting the biomechanical test, a pre-load of 5 N was applied. For the main testing series, the following set-up was used: following 10 settling cycles from 20 to 125 N, the bones were axially loaded from 20 to 500 N, respectively, in 5000 cycles with 25 mm/min. In a second testing series, the cyclic maximum load was increased to 1000 N. The cyclic loading levels with 500 and 1000 N represented the loading generated by a 90 kg person on the lateral tibial plateau during half and full weight bearing in daily life activity^18,19^, and were tested for applicability in a pre-series. Both cyclic loading levels were tested separately from each other with different specimens to analyse the initial depression (secondary subsidence) of the articular fracture fragment under loading. A load-to-failure test concluded the biomechanical test for all specimens. All data were recorded using testXpert II (Zwick GmbH & Co. KG, Ulm, Germany).

### Parameters of interest

Parameters of interest were displacement of the articular fracture fragment [mm], stiffness [N/mm] and maximum load [N]. Displacement was measured as the distance of the indentor between the initial bone contact at the start position and the unloaded position directly above the fragment after the cyclic loading phase. The stiffness was calculated at the beginning of the load–displacement curve of the load-to-failure tests, defined as the slope in the linear part of the curve. The normalized maximum load was further calculated by relating the absolute load to the initial maximum load of the native synthetic bone: The initial maximum load of the bone was determined as the maximum load, which was needed to produce the tibial head depression fracture in the fracture model of the synthetic bone. We calculated the normalized maximum load as the division of the maximum load in the load-to-failure tests and the initial maximum load of the native synthetic bone, to account for a possible batch difference.

### Statistical analysis

Sample size calculation was based on a previous study in which tibial head depression fractures were fixed with screws and screws with additional bone cement^9^. The displacement of the reduced fracture was measured after 3000 cycles of loading with 250 N. Within a statistical expert opinion by the Institute for Mathematics and Statistics of the University of Würzburg Cohen’s *d* calculated from the means and standard deviations of the mentioned two groups was 1.48 (group 1: Norian^®^ + screws, mean displacement ± S.D. = 1.1 mm ± 0.3 mm, group 2: screws only, mean displacement ± S.D. = 1.8 mm ± 0.6 mm). Setting $\beta$ at 0.2 and $\alpha$ at 0.05, a group size of n = 9 specimens per group was obtained. Moreover, conducting a post-hoc power analysis of the last biomechanical study from 2019 with a similar test set up, similar research hypothesis and n = 9 yielded a power of 99% for the displacement during the measuring cycles of the 7 different reduction groups^20^. Group size calculation was done with G*Power (Version 3.1.9.6, Heinrich Heine University Düsseldorf, Germany). Consequently, in all 14 groups of this study n = 9 was set.

The data of this study was first tested for normal distribution using the Shapiro–Wilk test and analysing QQ-Diagrams. Normal distributed data was then analysed with parametric statistics by using a one-way ANOVA. If a Levene-Test confirmed the homogeny of the variances in the normally distributed data, one-way ANOVA was used to determine differences in the means. These differences were then compared using a Tukey-HSD. If the homogeny of the variances could not be confirmed, a Welch-Test was used to determine differences between the groups. If this could be confirmed, the Dunnet-T3 test was used to compare the groups.

Not normally distributed data was analysed with non-parametric statistics using a Kruskal–Wallis-Test. If this showed differences between the data, a Mann–Whitney-U test with a Bonferroni Correction for multiple testing was used to compare the groups and determine statistically significant differences. The level of significance was set at p < 0.05.

The above-described data analysis was agreed with the Institute for Clinical Epidemiology and Biometry of the University Hospital of Würzburg and was performed using SPSS Statistics® (Version 23.0, IBM, Armonk, USA).

## Results

The results of all groups in both loading levels (500 and 1000 N) are shown in Table 2.

**Table 2:** The results of all parameters of interest are shown with the mean and standard deviation of every group.

Groups	Displacement settling + measuring cycles [mm]	Maximum load [N]	Normalized maximum load [%]	Initial fracture generation [N]	Stiffness [N/mm]
**500 N**					
1 (ChronOs)	6.5 ± 4.0	1788 ± 684	148 ± 64	1237 ± 196	495 ± 163
2 (2 Screws)	5.2 ± 0.7	2078 ± 207	201 ± 47	1075 ± 165	383 ± 43
3 (2 Screws + ChronOs)	3.7 ± 1.3	2990 ± 507	278 ± 37	1083 ± 173	520 ± 108
4 (4 Screws)	5.2 ± 0.7	2945 ± 251	257 ± 32	1160 ± 162	368 ± 97
5 (4 Screws + ChronOs)	6.2 ± 2.4	3085 ± 368	273 ± 70	1198 ± 189	516 ± 109
6 (Buttress Plate)	8.9 ± 2.8	2695 ± 665	222 ± 60	1228 ± 143	509 ± 73
7 (Buttress Plate + ChronOs)	3.1 ± 1.4	2832 ± 796	252 ± 139	1379 ± 200	792 ± 150
**1000 N**					
8 (ChronOs)	5.6 ± 2.2	2324 ± 304	218 ± 58	1120 ± 250	795 ± 112
9 (2 Screws)	7.9 ± 1.1	2268 ± 251	207 ± 32	1109 ± 135	549 ± 37
10 (2 Screws + ChronOs)	6.5 ± 1.2	2777 ± 361	254 ± 35	1102 ± 124	667 ± 92
11 (4 Screws)	8.9 ± 1.4	2636 ± 359	231 ± 61	1194 ± 270	624 ± 80
12 (4 Screws + ChronOs)	5.7 ± 0.8	3144 ± 237	245 ± 35	1306 ± 194	785 ± 145
13 (Buttress Plate)	16.9 ± 3.6	3024 ± 685	275 ± 62	1121 ± 204	892 ± 145
14 (Buttress Plate + ChronOs)	5.6 ± 1.3	2780 ± 699	285 ± 76	982 ± 77	916 ± 119

### Displacement

The results presented here are the total displacement values from the settling and measuring cycles (unloaded start position to unloaded end position). The displacement in the settling cycles was in all groups eminent, from a clinical perspective, and therefore taken into account.

**Figure 3:**
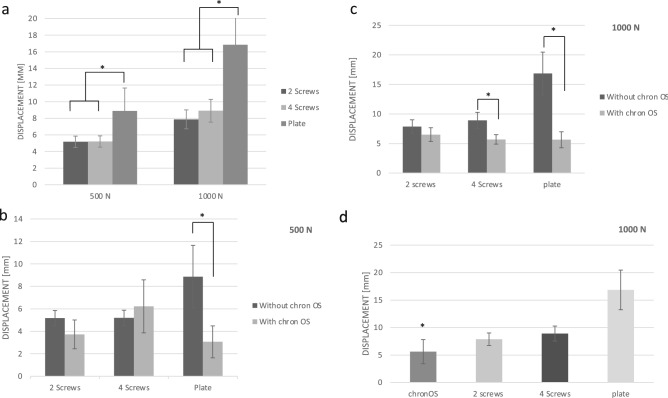
The results analysing the displacement of both loading levels is shown. Means ± SD, significant differences are indicated by *(p < 0.05 between groups). (**a**) In both loading levels, the plate osteosynthesis revealed a higher displacement compared to the screw osteosyntheses. (**b**) Comparing the different types of osteosyntheses alone and in combination with bone cement to each other, a significant lower displacement was determined for plate osteosynthesis in the 500 N loading level. (**c**) In the 1000 N testing series, a lower displacement was detected for the combination with bone cement for the 4 screw and the plate osteosynthesis. (**d**) The group with bone filler only (ChronOS™ inject) revealed a lower displacement in the 1000 N loading level compared to the three types of osteosyntheses only.

The plate osteosynthesis revealed a higher displacement regardless of whether cement was used or not compared to both screw osteosyntheses (group 2, 6/group 4, 6/group 9, 13/group 11, 13 [p < 0.01]) (Fig. 3a). Comparing the osteosyntheses all in combination with bone cement to each other, the plate showed a trend to a lower displacement, but the significance levels were not reached (500 N: group 3, 5 [p = 0.90]; group 3, 7 [p = 0.64]; group 5, 7 [p = 0.92]) (1000 N: group 10, 12 [p = 0.11]; group 10, 14 [p = 0.14]; group 12, 14 [p = 0.86]).

No significant differences were determined comparing the screw osteosyntheses alone and in combination with bone cement in the testing series with 500 N (group 2, 3 [p = 0.48]; group 4, 5 [p = 0.82]). For the plate osteosynthesis, a significant lower displacement was determined for the combination with ChronOS™ inject (group 6, 7 [p < 0.01]) (Fig. 3b). In the testing series with 1000 N, a significant difference for the 4 screw and the plate osteosynthesis with and without bone cement were detected (group 9, 10 [p = 0.78], group 11, 12 [p = 0.01]; group 13, 14 [p < 0.01]) (Fig. 3c).

For the control group with bone cement only, there were no differences in-between groups in the 500 N level testing series. Interestingly, for the 1000 N level, a significant lower displacement for the ChronOS™ inject only group compared to the three types of osteosyntheses without bone cement was demonstrated (group 8, 9 [p = 0.04]; group 8, 11 [p < 0.01]; group 8, 13 [p < 0.01] (Fig. 3d).

### Stiffness

**Figure 4:**
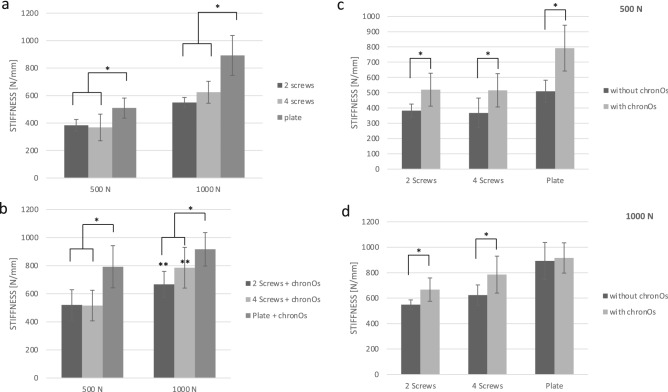
The results of the stiffness are shown. Means ± SD, significant differences are indicated by *(p < 0.05 between groups). (**a**) A higher stiffness revealed the plate osteosynthesis compared to the two screw osteosyntheses in both loading levels. (**b**) The plate exhibited a higher stiffness compared to the screw osteosyntheses in both loading levels when combined with cement. Additionally, in the 1000 N level, the 4 screws with ChronOs™ revealed a higher stiffness compared to the 2 screws with ChronOs™ (indicated by **). (**c**) A higher stiffness revealed the combination with bone cement compared to the same type of osteosynthesis alone in the 500 N loading level. (**d**) For the two screw osteosyntheses a higher stiffness was determined for the combination with bone cement compared to the same type of osteosynthesis without bone filler in the 1000 N loading level.

The plate osteosynthesis revealed a higher stiffness of whether cement was used or not compared to both screw osteosyntheses (group 2, 6/group 4, 6/group 9, 13/group 11, 13 [p < 0.01]) (Fig. 4a). The plate osteosynthesis in combination with bone cement determined in both loading levels a significantly higher stiffness compared to the two screw osteosyntheses in combination with bone cement (group 3, 7/group 5, 7/group 10, 14/group 12, 14 [p < 0.01]). For the 1000 N testing series, additionally a higher stiffness could be detected for 4 screws compared to the 2 screws, both with bone filler (group 10, 12 [p = 0.03]) (Fig. 4b).

In both loading levels, the osteosyntheses demonstrated a significantly higher stiffness for the combination with bone cement compared to the osteosyntheses alone (500 N: group 2, 3 [p < 0.01]; group 4, 5 [p < 0.01]; group 6, 7 [p < 0.01) (1000 N: group 9, 10 [p = 0.01]; group 11, 12 [p = 0.01]). Only the plate osteosynthesis revealed no significant difference in the 1000 N level with and without bone filler (group 13, 14 [p = 0.67]) (Fig. 4c+d).

### Maximum load

**Figure 5:**
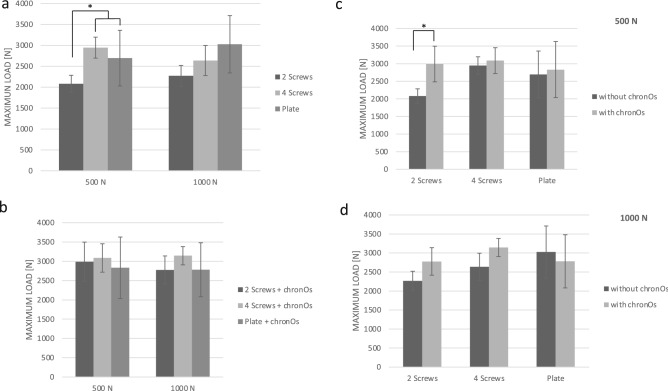
The results of the maximum load are demonstrated. Means ± SD, significant differences are indicated by *(p < 0.05 between groups). (**a**) A higher maximum load could be detected for the 4 screw and the plate osteosynthesis in the 500 N loading level compared to the 2 screw osteosynthesis, all without bone filler. (**b**) Comparing the three types of osteosynthesis with bone cement to each other, no differences were distinguished. (**c**) A higher maximum load for the combination with ChronOs™ inject revealed the two screw osteosynthesis whereas no differences were determined for the 4 screw and the plate osteosynthesis in the 500 N level. (**d**) No differences were detected between the different types of osteosyntheses without bone cement compared to the same type in combination with bone cement in the 1000 N testing series.

Comparing the three types of osteosyntheses alone (without bone filler) to each other, in the 500 N level the 2 screw osteosynthesis revealed a lower maximum load than the 4 screw osteosynthesis and the plate osteosynthesis (500 N: group 2, 4 [p < 0.01]; group 2, 6 [p = 0.03]; group 4, 6 [p = 0.08]). No differences for the osteosyntheses were detected in the 1000 N testing series (1000 N: group 9, 11 [p = 0.43]; group 9, 13 [p = 0.36]; group 11, 13 [p = 0.25]) (Fig. 5a).

For the three types of osteosyntheses in combination with bone cement, no differences were determined in both loading levels (500 N: group 3, 5 [p = 0.17]; group 3, 7 [p = 0.34]; group 5, 7 [p = 0.13]) (1000 N: group 10, 12 [p = 0.42]; group 10, 14 [p = 0.11]; group 12, 14 [p = 0.27]) (Fig. 5b).

Whilst a general trend to a higher maximum load could be detected for the combination of osteosynthesis with bone cement compared to the same osteosynthesis alone, the significance level was not reached in both loading levels (500 N: group 4, 5 [p = 0.16]; group 6, 7 [p = 0.80]) (1000 N: group 9, 10 [p = 0.06]; group 11, 12 [p = 0.06]; group 13, 14 [p = 1.00]) (Fig. 5c+ d). Except, for the 2 screws tested in the 500 N level, a significance difference for the combination with bone cement compared to the osteosynthesis alone could be detected (group 2, 3 [p < 0.01]) (Fig. 5c).

The analysis of the normalized maximum load did not reveal diverging differences between the groups than the absolute values for the maximum load. The initial load at fracture generation did not differ significantly between groups (Table 2).

### Mode of failure

For the group with the two screws in combination with the ChronOS™ Inject, the mode of failure was determined to be the load under which the screws failed. For the four screws, it was determined at the load under which the two smaller screws failed. In the plate osteosynthesis group, the mode of failure was determined as the load under which the bone of the tibial plateau failed and the tibial shaft developed a fracture at the distal plate end. The osteosynthesis construct itself did not give way.

## Discussion

Considering the problems seen in clinical practice when treating older patients, especially higher loading of the tibial plateau postoperatively than desired by the surgeon, studies analysing differences in stability of different osteosyntheses under high loading conditions are needed. To date, to our knowledge, there are only a few biomechanical studies in which higher loading conditions of the tibial plateau were simulated to better understand problems associated with treating tibial head fractures. For example, McDonald et al.^14^ compared autologous bone graft to bone cement under higher loading conditions in split depression tibial plateau fractures, demonstrating biomechanical advantages for the bone cement. Benoit et al.^15^ analysed a new option of filling the metaphyseal bone defect with trabecular metal, revealing that a higher stability was achieved with this technique compared to an impacted cancellous bone graft in tibial head depression fractures. Furthermore, Blakey et al.^16^ simulated higher loading conditions when investigating an interference screw as an osteosynthesis alone in pure depression fractures of the tibial plateau. Under high loading conditions, low stability was found for the interference screw alone why using this osteosynthesis alone is not reasonable.

The options used in this study for stabilization of the simulated fractures are well-established techniques in tibial head fractures. In pure split or pure depression fractures, a 2 screw osteosynthesis with cancellous screws is often applied. A combination of 3 to 4 screws arranged in the jail technique is also described for these tibial head fractures in different ways as 6.5 mm cancellous screws with 4.0 mm small fragment screws or 3.5 mm cortical screws^7–9,21^. In unicondylar tibial head fractures, buttress plates usually with angle-stable screws are used when the type of the tibial head fracture demands a higher stability than a screw osteosynthesis alone like for example in split depression fractures.

Former biomechanical studies applying comparatively low loading analysed the influence of the type of osteosynthesis on the stability and showed advantages for the screws in the jail technique compared to only 2 screws^7,8,22^. Furthermore, the screws in the jail technique also provided an equivalent high stability in combination with bone cement compared to the plate osteosynthesis under maximal loading conditions. Simulating a usual partial weightbearing with 20 kg, the jail technique demonstrated the lowest subsidence of the articular fracture fragment compared to the 2 screws and the plate^22^. In contrast, in this study, with higher loading of the tibial plateau, the plate osteosynthesis in combination with bone cement revealed a trend to the highest resistance against a loss of reduction in the cyclic loading phase (the lowest absolute displacement, however not statistically significant). Thus, under higher loads, a more stable osteosynthesis like the angle-stable buttress plate has biomechanical advantages compared to screw osteosyntheses. In addition, the plate in combination with bone cement demonstrated a higher stiffness compared to all other groups. It seems that the biomechanical advantages of the plate are more evident under maximal loading, when already higher loading conditions were performed previously (in the cyclic loading phase) in contrast to the results of a former study with lower cyclic loading conditions^22^. The use of a plate instead of screws in pure tibial head depression fractures must be discussed, however, still critically considering the more extensive necessary soft tissue preparation, even when there are biomechanical advantages demonstrated for higher loading. This study can only provide information about the in vitro conditions; the type of osteosynthesis used intraoperatively must still be discussed from case to case.

The filling up of the metaphyseal bone defect is essential to reduce the secondary loss of reduction of the articular fracture fragment under lower loading conditions like shown in a previous study^9^ as well as in this study under higher loading conditions. At the 1000 N level the ChronOs™ only group demonstrated the significant lowest displacement when compared to the other osteosyntheses without cement. This is likely due to a more precise filling of the metaphyseal defect as no hindering screws were in the way. In all types of osteosyntheses, the use of bone cement resulted in a higher stiffness compared to the osteosyntheses alone. Interestingly, significant differences in the subsidence of the articular fracture fragment (displacement) for the combination of bone cement and the osteosyntheses were only seen in the 1000 N loading level. These results are well in agreement with a former biomechanical study with lower cyclic loading conditions^22^. On loading level 500 N, however, no differences for the screw osteosyntheses with and without bone cement were detected. It might be, that under higher loading conditions, the osteosyntheses are more important for the reduction of the displacement than under lower loading conditions.

The use of a calcium phosphate bone filler like applied in this study is well established in daily clinical work and associated with a higher stability compared to crest bone graft in biomechanical studies^12,23^.

Interestingly, the bone cement used had no influence on the mode of failure. The screw construct was found to be the limiting factor in this study. In the osteosynthesis group, the locking plate and screws themselves did not fail. Instead, the bone gave way around the implant as the load increased.

It has to be noted that the biomechanical test set-up used for this study reduced the acting forces on the tibial plateau to the main axial loading forces, and here on the lateral part of the plateau. However, even if this set-up cannot completely simulate the physiological conditions, it compares well with former biomechanical studies using similar test set-ups^12,14–16,24^, and this study contributes important biomechanical information about the differences in stability of several treatment options. We also recognize that the use of the indentor loaded exclusively the reduced fracture fragment and therefore created a more rigorous test set-up as an artificial knee joint as load applicator would have done. This eliminated possible disruptive factors. The use of a synthetic bone instead of a human bone may be regarded as another limitation, however, like demonstrated before, this special type of synthetic bone resembles very well the biomechanical qualities of a human osteoporotic tibial head^9,10^, and moreover, an additional advantage of the use of synthetic bones is a low interspecimen variation.

## Conclusions

Under higher loading conditions, a plate osteosynthesis in combination with a bone cement provides the highest stability compared to screw osteosyntheses and should be considered for fracture stabilization in special patient cases of pure depression fractures, when a postoperative high loading is likely, and the risk of a more extensive operative technique is justifiable.

## Data Availability

The test records for all specimens can be obtained from the corresponding author upon reasonable request.
